# The effect of posture on virtual walking experience using foot vibrations

**DOI:** 10.1038/s41598-024-70229-5

**Published:** 2024-08-21

**Authors:** Junya Nakamura, Michiteru Kitazaki

**Affiliations:** Department of Computer Science and Engineering, Toyohashi University of Technology, 1-1 Hibarigaoka, Tempaku, Toyohashi, Aichi 441-8580 Japan

**Keywords:** Walking, Posture, Virtual reality, Sensory integration, Human behaviour, Computer science

## Abstract

Virtual walking systems for stationary observers have been developed using multimodal stimulation such as vision, touch, and sound to overcome physical limitation. In previous studies, participants were typically positioned in either a standing or a seated position. It would be beneficial if bedridden users could have enough virtual walking experience. Thus, we aimed to investigate the effects of participants’ posture and foot vibrations on the experience of virtual walking. They were either sitting, standing, or lying during observing a virtual scene of a walking avatar in the first-person perspective, while vibrations either synchronized or asynchronized (randomized) to the avatar’s walking were applied to their feet. We found that the synchronized foot vibrations improved virtual walking experiences compared to asynchronous vibrations. The standing position consistently offered an improved virtual walking experience compared to sitting and lying positions with either the synchronous or asynchronous foot vibrations, while the difference between the siting and lying postures was small and not significant. Furthermore, subjective scores for posture matching between real and virtual postures, illusory body ownership, and sense of agency were significantly higher with the synchronous than the asynchronous vibration. These findings suggest that experiencing virtual walking with foot vibrations in a lying position is less effective than a standing position, but not much different from a sitting position.

## Introduction

Walking represents a fundamental human activity, influencing not only physical mobility but also cognitive and sensory perceptions. The simulation of walking in virtual environments using Virtual Reality (VR) and Augmented Reality (AR) has garnered significant interest, posing a challenge to replicate real-world walking sensations. Research on movement in cyberspaces is rapidly advancing with the emergence of the metaverse. Virtual walking shows great potential for a variety of applications, including tourism, entertainment, training, and rehabilitation.

Walking is known to be semi-automatically controlled, primarily by the central pattern generator (CPG)^1^. Additionally, our locomotion is optimized through the integration of sensory information such as vision, vestibular senses, and proprioception. This process of sensory integration is dynamically weighted based on the reliability and importance of each sensory input^2–4^. For example, when a particular sense is deemed unreliable, its weight diminishes accordingly, as indicated by various studies^5–7^. The process of sensory integration can vary depending on an individual’s experiences, intentions, and circumstances, leading to variations in the quality and perception of walking^8,9^.

Vision is one of important sensations during walking. The visually-induced sensation of self-motion is generally known as vection^10–12^. The induction of vection becomes more effective under specific conditions. It is enhanced with motion presented across a large visual field^10,13^. The motion of the background dominates over that of the foreground^14,15^. Non-attended, rather than attended motion determines vection direction^16^. Vection is enhanced by perspective jitter^17,18^. Non-visual modality information, such as sound and touch affect illusory self-motion perception^19,20^. It is facilitated by naturalistic and globally consistent stimuli^21,22^. The utilization of vection in virtual walking systems has become increasingly popular, as the impression of self-motion can be provided without any physical movement^23–26^. Integrating simulated camera motions (similar to perspective jitter) with the vection stimulus, such as an expanding radial flow, improves the walking sensation of seated users^23^.

Adding tactile feedback to vection amplifies the realistic sensation of walking in virtual environments^24–26^. Notably, Kitazaki et al.^26^ elucidated that foot vibrations synchronized with optic flow not only enhance the sense of self-motion but also the sensations of walking, leg movement, and telepresence. Telepresence refers to the sensation of being present in a virtual or remote environment and is deemed an essential element in deepening the VR experience^27^. Koilias et al.^28^ demonstrated that specific tactile feedback conditions significantly influence movement behavior and self-reported realism during immersive VR walking experiences. The multisensory VR system developed by Turchet et al.^29^ enhances the realism of virtual walking by providing visual scenes, footsteps sounds, and tactile feedback to the feet based on the type of terrain. This system incorporates walking and running avatars viewed from a third-person perspective. While the study suggests that multisensory congruence enhances the realism of walking, it does not explicitly evaluate the effect of the avatar itself.

Experiencing tactile sensations synchronized with an avatar can induce an illusory sense of body ownership^30–33^. When the avatar moves in synchronization with the user, the illusory body ownership becomes more pronounced^34,35^. The sense of agency refers to the subjective experience of controlling one’s actions or events in a virtual world^36,37^. Kokkinara et al.^38^ demonstrated that users can experience an illusory sense of body ownership and agency for a walking avatar in a virtual environment, even while the participants are physically seated. However, they used only the visual sensation without tactile stimulation. Matsuda et al.^39^ clarified that observing the walking of a self-body avatar in the first person perspective with receiving synchronous vibrations on the feet contributes to enhancing the sensation of walking. Thus, the multistory information such as optic flow and tactile sensation to the feet induces virtual walking sensation with embodiment, which is enhanced by observing the self avatar from the first person perspective. Moreover, Saint-Aubert et al.^40^ has shown that vibrotactile feedback on the feet improves the impression of walking and embodiment in both first and third-person perspectives.

Recent studies have expanded understanding of various factors influencing virtual walking experiences. Mousas et al.^41^evaluated VR locomotion interfaces, suggesting that joysticks and walk-in-place provided more realistic experiences than omnidirectional treadmills. Simeone et al.^42^ investigated how altering the visual representation of surfaces and introducing immaterial objects in virtual environments affects users’ movement patterns, providing design guidelines for intentionally altering user movement behavior. In another study, Mousas et al.^43^ revealed that mismatches between real and virtual environments significantly alter psychological and movement responses during virtual walking. Lynch et al.^44^ observed that body motion cues alone were sufficient for navigation in virtual environments, with gaze behavior having minimal impact. Janeh et al.^45^ revealed that discrepancies between real and virtual walking velocities significantly affect gait stability in VR. Cirio et al.^46^ proposed a framework for evaluating the realism of walking trajectories in virtual reality. Soczawa-Stronczyk et al.^47^ demonstrated that while VR can effectively simulate real-world walking behaviors, minor quantitative differences in gait coordination exist between the virtual and real environments. Multon et al.^48^ provided detailed biomechanical data to enhance the naturalness of walking experiences in VR environments. These studies collectively contribute to our understanding of virtual walking experiences and highlight the complex interplay of visual, tactile, and environmental factors in creating realistic and immersive VR walking simulations.

Technological advances and research have enabled the creation of virtual walking experiences with postures distinct from actual walking. User demand for VR experiences with fewer physical limitations that enable more freedom of movement is increasing. A larger number of experiments are being conducted to explore virtual walking while seated^26,38,39^. However, currently there is insufficient research examining the discrepancy experienced between the actual walking posture and the observation posture in VR. Research by Saint-Aubert et al.^49^ investigated the influence of body posture on the sensation of walking. Their results showed that the sensation of walking and the sense of body ownership in Fowler’s posture (a reclined supine position with the backrest of the trunk tilted about 45 degree) was weaker compared to when standing or sitting. Their research primarily focused on the influence of visual stimuli, and the effects of a multisensory combination of visual and tactile stimuli have not been clarified.

Therefore, this study focuses specifically on the impact of the combination of posture and foot vibration on the walking experience in VR, particularly in the situation where walking is just observed virtually without any movements of body such as legs or arms. Four hypotheses were proposed. Foot vibration stimuli synchronized with walking enhances the sensation of walking (H1a) and the sense of embodiment (H1b) independent of posture. These are consistent with an existing study by Saint-Aubert et al.^40^; Tactile sensation on the feet with virtual feet hitting the ground increases the sense of ownership of a walking virtual avatar. It can reduce the disparity between real and virtual posture. The lying position has the advantage in posture similarity of joints, while the sitting posture has the advantage in gravity similarity for human vestibular system to the actual walking. The sensation of walking and embodiment from visual stimulation in Fowler’s posture similar to lying position is weaker than seated posture^49^. However, the walking-simulating vibrations to the soles of the feet may reduce the discrepancy of gravity in the lying position. Therefore, we hypothesized that the walking sensation (H2a) and the embodiment sensation (H2b) in the lying position would be equivalent to those in the seated position when the synchronous vibrations are applied to the feet. Participants observed visual stimuli of virtual walking while receiving either synchronous or asynchronous foot vibrations and rated walking sensation, telepresence, embodiment perception, and VR sickness. Their posture during the experiments was either standing, sitting, or lying. Both factors (foot vibration and posture) were within-participant variables.

## Results

First, we tested the normality of the all questionnaire data (Shapiro–Wilk test, alpha = 0.05). If the data did not violate the normality test (p > 0.05), a two-way repeated measures ANOVA was performed. If the data violate the normality test (p < 0.05), a two-way repeated measures ANOVA with an aligned rank transformation (ANOVA with ART)^50^ was performed as non-parametric test. Then, we conducted an analysis of simple main effects and a post-hoc multiple-comparison analysis as necessary. For parametric data, if there was a lack of sphericity with Mendoza’s multisample sphericity test, the reported values were adjusted using the Greenhouse-Geisser correction^51^. Shaffer’s modified sequentially rejective Bonferroni procedure was applied for post-hoc multiple comparisons. For non-parametric data, Tukey’s method with Kenward–Roger degrees of freedom approximation^52^ was applied for post-hoc multiple comparison analysis.

### Walking-related sensations and telepresence

**Figure 1:**
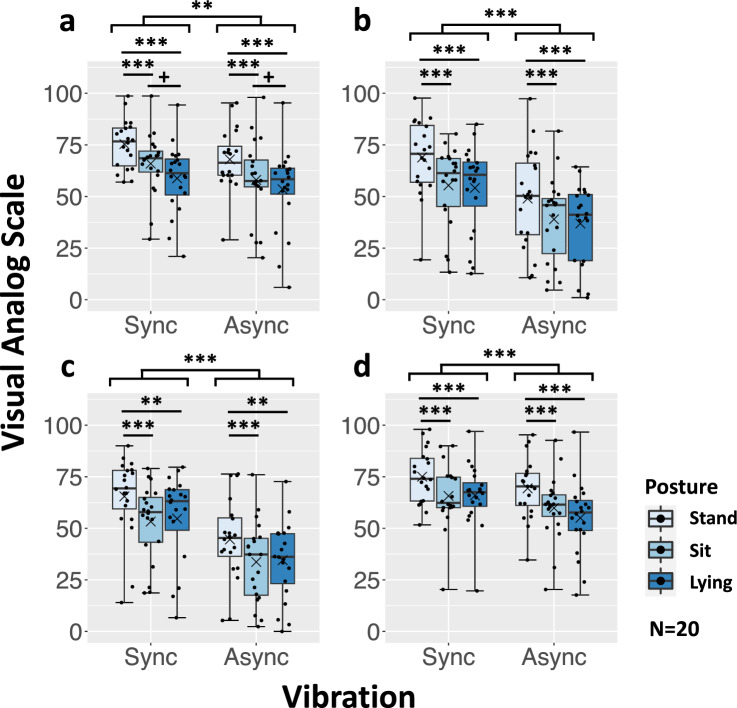
Results of walking-related sensations and telepresence: (**a**) Self-motion. (**b**) Walking sensation. (**c**) Leg action sensation. (**d**) Telepresence. The thick line in the box indicates the median, and a set of whiskers indicates the maximum and minimum values. The dots indicate the dispersion of scores, and the cross (x) indicates the mean value. ***p < 0.001, **p < 0.01, *p < 0.05, $^{+}$*p* < 0.1.

Since data of all items violated normality, we conducted a two-way repeated measures ANOVA with ART. The statistical results were basically identical across all items, as follows (Fig. 1).

#### Self-motion sensation

For self-motion sensation, we found a significant main effect for the foot vibrations [*F*(1,19) = 13.13, *p* = 0.002, $\eta _{p}^{2}$ = 0.41] and the posture [*F*(2,38) = 22.28, *p* < 0.0001, $\eta _{p}^{2}$ = 0.54], but the interaction was not significant [*F*(2,38) = 0.767, *p* = 0.471, $\eta _{p}^{2}$ = 0.039] (Fig. 1a). The score was significantly higher with the synchronous than the asynchronous foot vibration. The post-hoc analysis of the posture conditions showed that the scores for the standing posture were significantly higher than the sitting posture [*t*(38) = 4.17, adj.*p* = 0.0005, coh.d = 1.18] and lying posture [*t*(38) = 6.60, adj.*p* < 0.0001, coh.d = 1.86]. There was no significant difference between the sitting and lying postures [*t*(38) = 2.42, adj.*p* = 0.052, coh.d = 0.683].

#### Walking sensation

For walking sensation, we found a significant main effect for the foot vibrations [*F*(1,19) = 52.40, *p* < 0.0001, $\eta _{p}^{2}$ = 0.73] and the posture [*F*(2,38) = 15.69, *p* < 0.0001, $\eta _{p}^{2}$ = 0.45], but the interaction was not significant [*F*(2,38) = 0.325, *p* = 0.725, $\eta _{p}^{2}$ = 0.017] (Fig. 1b). The score was significantly higher with the synchronous than the asynchronous foot vibration. The post-hoc analysis of the posture conditions showed that the scores for the standing posture were significantly higher than the sitting posture [*t*(38) = 4.52, adj.*p* = 0.0002, coh.d = 1.24] and lying posture [*t*(38) = 5.13, adj.*p* < 0.0001, coh.d = 1.41]. There was no significant difference between the sitting and lying postures [*t*(38) = 0.611, adj.*p* = 0.815, coh.d = 0.168].

#### Leg-action sensation

For leg-action sensation, we found a significant main effect for the foot vibrations [*F*(1,19) = 71.57, *p* < 0.0001, $\eta _{p}^{2}$ = 0.79] and the posture [*F*(2,38) = 12.57, *p* < 0.0001, $\eta _{p}^{2}$ = 0.40], but the interaction was not significant [*F*(2,38) = 0.318, *p* = 0.729, $\eta _{p}^{2}$ = 0.016] (Fig. 1c). The score was significantly higher with the synchronous than the asynchronous foot vibration. The post-hoc analysis of the posture conditions showed that the scores for the standing posture were significantly higher than the sitting posture [*t*(38) = 4.72, adj.*p* < 0.0001, coh.d = 1.35] and lying posture [*t*(38) = 3.82, adj.*p* = 0.001, coh.d = 1.09 ]. There was no significant difference between the sitting and lying postures [*t*(38) = 0.903, adj.*p* = 0.642, coh.d = 0.259].

#### Telepresence sensation

For telepresence sensation, we found a significant main effect for the foot vibrations [*F*(1,19) = 18.79, *p* = 0.0004, $\eta _{p}^{2}$ = 0.50] and the posture [*F*(2,38) = 17.30, *p* < 0.0001, $\eta _{p}^{2}$ = 0.48], but the interaction was not significant [*F*(2,38) = 2.087, *p* = 0.138, $\eta _{p}^{2}$ = 0.099] (Fig. 1d). The score was significantly higher with the synchronous than the asynchronous foot vibration. The post-hoc analysis of the posture conditions showed that the scores for the standing posture were significantly higher than the sitting posture [*t*(38) = 4.56, adj.*p* = 0.0002, coh.d = 1.33] and lying posture [*t*(38) = 5.50, adj.*p* < 0.0001, coh.d = 1.607]. There was no significant difference between the sitting and lying postures [*t*(38) = 0.941, adj.*p* = 0.612, coh.d = 0.275].

### Embodiment score

**Figure 2:**
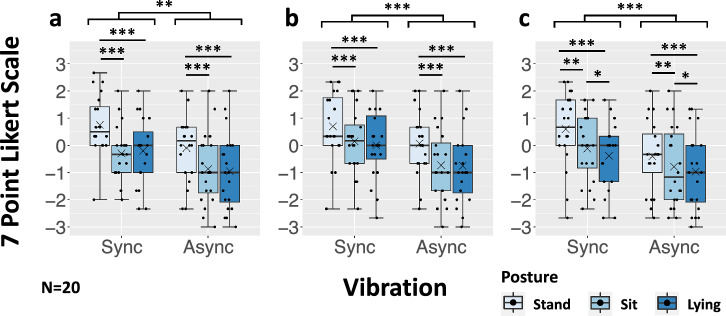
Results of embodiment scores: (**a**) Posture matching. (**b**) Body ownership. (**c**) Sense of agency. The thick line in the box indicates the median, and a set of whiskers indicates the maximum and minimum values. The dots indicate the dispersion of scores, and the cross (x) indicates the mean value. ***p < 0.001, **p < 0.01, *p < 0.05.

Since data of all items did not violate normality, we conducted a two-way repeated measures ANOVA. The statistical results were basically identical across all items, as follows (Fig. 2).

#### Posture matching

For posture matching score, we found a significant main effect for the foot vibrations [*F*(1,19) = 15.22, *p* = 0.001, $\eta _{p}^{2}$ = 0.44] and the posture [*F*(2,38) = 15.95, *p* < 0.0001, $\eta _{p}^{2}$ = 0.46], but the interaction was not significant [*F*(2,38) = 0.934, *p* = 0.402, $\eta _{p}^{2}$ = 0.047] (Fig. 2a). The score was significantly higher with the synchronous than the asynchronous foot vibration, indicating that the participants felt less discrepancy of posture with the synchronous foot vibration compared with the asynchronous vibration. The post-hoc analysis of the posture conditions showed that the scores for the standing posture were significantly higher than the sitting posture [*t*(19) = 4.61, adj.*p* = 0.0006, coh.d = 0.750] and lying posture [*t*(19) = 4.32, adj.*p* = 0.0006, coh.d = 0.743]. There was no significant difference between the sitting and lying postures [*t*(19) = 0.059, adj.*p* = 0.954, coh.d = 0.007].

#### Body ownership

For body ownership score, we found a significant main effect for the foot vibrations [*F*(1,19) = 26.26, *p* = 0.0001, $\eta _{p}^{2}$ = 0.58] and the posture [*F*(2,38) = 18.27, *p* < 0.0001, $\eta _{p}^{2}$ = 0.49], but the interaction was not significant [*F*(2,38) = 0.947, *p* = 0.397, $\eta _{p}^{2}$ = 0.048] (Fig. 2b). The participants felt higher illusory body ownership with the synchronous foot vibration compared with the asynchronous vibration. The post-hoc analysis of the posture conditions showed that the scores for the standing posture were significantly higher than the sitting posture [*t*(19) = 5.60, adj.*p* = 0.0001, coh.d = 0.535] and lying posture [*t*(19) = 4.64, adj.*p* = 0.0002, coh.d = 0.601]. There was no significant difference between the sitting and lying postures [*t*(19) = 0.668, adj.*p* = 0.512, coh.d = 0.066].

#### Sense of agency

For sense of agency score, we found a significant main effect for the foot vibrations [*F*(1,19) = 18.78, *p* = 0.0004, $\eta _{p}^{2}$ = 0.50] and the posture [*F*(1.63, 30.97) = 15.44, *p* = 0.0001, $\eta _{p}^{2}$ = 0.45], but the interaction was not significant [*F*(2,38) = 2.333, *p* = 0.111, $\eta _{p}^{2}$ = 0.11] (Fig. 2c). The participants felt higher sense of agency with the synchronous foot vibration compared with the asynchronous vibration. The post-hoc analysis of the posture conditions showed that the scores for the standing posture were significantly higher than the sitting posture [*t*(19) = 3.66, adj.*p* = 0.002, coh.d = 0.410] and lying posture [*t*(19) = 4.63, adj.*p* = 0.0005, coh.d = 0.577], and the scores for the sitting posture were significantly higher than the lying posture [*t*(19) = 2.12, adj.*p* = 0.048, coh.d = 0.168].

### Virtual Reality Sickness Questionnaire (VRSQ)

**Figure 3:**
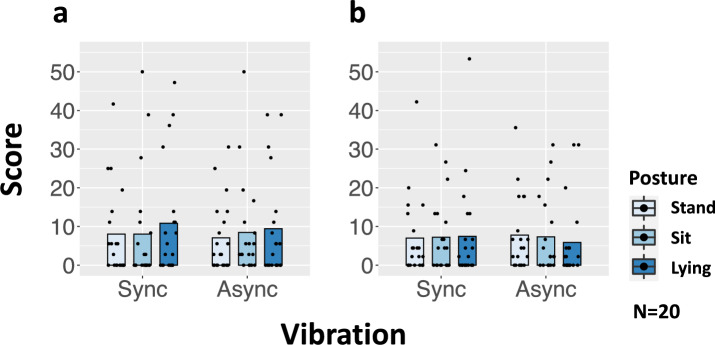
Results of Virtual Reality Sickness Questionnaire (VRSQ) scores: (**a**) Oculomotor. (**b**) Disorientation. The dots indicate the dispersion of scores, and the bar indicates the mean score.

Based on the VR Sickness Questionnaire, the data for the Oculomotor and Disorientation indicators did not satisfy the normality. Thus, we conducted a two-way repeated measures ANOVA-ART. For the Oculomotor score, we did not find significant effects of the foot vibrations [*F*(1,19) = 3.35, *p* = 0.083, $\eta _{p}^{2}$ = 0.15] the posture [*F*(2,38) = 0.511, *p* = 0.603, $\eta _{p}^{2}$ = 0.026], or the interaction [*F*(2,38) = 2.62, *p* = 0.086, $\eta _{p}^{2}$ = 0.12] (Fig. 3a). For Disorientation score, our analysis revealed no significant main effect of the foot vibration [*F*(1,19) = 0.338, *p* = 0.568, $\eta _{p}^{2}$ = 0.017], the posture [*F*(2,38) = 1.49, *p* = 0.239, $\eta _{p}^{2}$ = 0.072] or the interaction [*F*(2,38) = 1.91, *p* = 0.162, $\eta _{p}^{2}$ = 0.091] (Fig. 3b). As an exploratory analysis, we performed the identical statistical tests for the dataset excluding the data of the top 25% (5 participants) with the strongest ratings of VR sickness (N=15). For the Oculomotor score, we found a significant effect of the foot vibrations [*F*(1,14) = 5.61, *p* = 0.033, $\eta _{p}^{2}$ = 0.286], but the main effect of the posture [*F*(2,28) = 0.140, *p* = 0.870, $\eta _{p}^{2}$ = 0.010] or the interaction [*F*(2,28) = 0.440, *p* = 0.649, $\eta _{p}^{2}$ = 0.030] was not significant. For Disorientation score, our analysis revealed no significant main effect of the foot vibration [*F*(1,14) = 1.18 *p* = 0.297, $\eta _{p}^{2}$ = 0.077], the posture [*F*(2,28) = 2.27, *p* = 0.122, $\eta _{p}^{2}$ = 0.139] or the interaction [*F*(2,28) = 0.852, *p* = 0.437, $\eta _{p}^{2}$ = 0.057]

## Discussion

### Summary of results

We investigated the effects of posture and foot vibrations on the experience of virtual walking. We observed that synchronized foot vibrations notably improved all four sensations (self motion, walking, leg action, and telepresence) compared to asynchronous vibrations. Moreover, the standing position consistently offered an improved virtual walking experience compared to sitting and lying positions, while the difference between the siting and lying postures was small and not significant in both synchronous and asynchronous vibration conditions. These findings support the hypothesis H1a that synchronized foot vibrations enhance the virtual walking experience. Furthermore, scores measuring subjective posture matching, body ownership, and sense of agency known as embodiment scores were significantly higher with the synchronous than the asynchronous vibration, thus supporting H1b. Either with the synchronous or asynchronous vibrations, the sense of body ownership and subjective posture matching for the lying posture were similar to the sitting posture without significant difference, while the sense of agency was significantly higher in the sitting than the lying posture.

### Effect of Foot vibration stimuli synchronized with walking (H1)

The effect of synchronous and asynchronous foot vibration was statistically significant in both walking evaluation and embodiment evaluation. Therefore, two hypotheses were supported; The walking experience by synchronized vibration improves the sense of walking (H1a) and improves embodiment (H1b) regardless of the observation posture. In addition to visual movement information, the rhythmic foot vibrations, combined with the vision of walking avatar, could simulate the leg motion during walking, so that the virtual walking experience occurred. Actually, the effect sizes of the sense of leg action ($\eta _{p}^{2}$ = 0.79) and the sense of walking ($\eta _{p}^{2}$ = 0.73) between synchronous and asynchronous vibration conditions were higher than the sense of self motion ($\eta _{p}^{2}$ = 0.41). The improvement of walking sensation by synchronous vibration has been obtained in previous studies^26,29^, and the present study showed that it is effective in different observation postures. Furthermore, the evaluation of embodiment (posture consistency, physical ownership, and agency) was also enhanced by synchronous foot vibration that was presented to participants’ foot synchronously with the avatar’s foot striking the ground. Thus, it could be caused by the visual-tactile integration such as the rubber-hand illusion^30^.

### Effects of observation posture on walking sensation and embodiment ratings (H2)

The results regarding the effects of observation posture with foot vibrations showed that there was no significant difference between the sitting and lying positions for self-motion, walking, and leg-action sensations, although the score was slightly higher in the sitting position, in both the synchronous and asynchronous vibration conditions. In the embodiment revaluation, sense of agency was significantly higher in the sitting position than in the lying position, again in both the synchronous and asynchronous vibration conditions. No significant difference was found for posture matching and body ownership scores.

Hypothesis H2 stated that the sitting and lying positions would be equivalent with synchronized foot vibrations. However, the consistently higher ratings of walking experience and embodiment in the seated position compared to the lying position, although not statistically significant, suggest that they cannot be considered equivalent. Therefore, H2a and H2b were not supported. Instead, the results suggest that tactile stimulation of the soles of the feet, independent of its synchrony with walking, could reduce the difference between the lying and sitting positions for the walking sensation and the embodiment except for the sense of agency.

The standing position, being the most akin to actual walking and serving as a reference, showed significant and highest sensations of walking and embodiment when compared to both sitting and lying positions. While the lying position aligns with the standing position in terms of posture similarity, the sitting position aligns with the direction of gravity for the vestibular system, resulting in a trade-off in their respective significances. This appears to have produced our results. However, there are concerns about claiming equivalence between the two, and further large-scale experiments are needed to test this equivalence.

### Evaluation of virtual reality sickness in posture and foot-vibration combinations

We evaluated the Virtual Reality Sickness Questionnaire (VRSQ) scores across all combinations of posture and foot vibration conditions. The Oculomotor and Disorientation scores showed no significant effect either between posture conditions or between foot vibration conditions. Each of these scores was around 10, indicating a low level and suggesting that this experiment did not induce severe virtual reality (VR) sickness. An exploratory analysis excluding the top 25% of severely sickened participants showed that synchronous foot vibrations reduced oculomotor scores. Taps (light force) to the head^53^, vibrations to the back of the ears^54^, or vibrations to the feet^55^ synchronized to the walking footsteps reduce VR sickness. It is suggested that the incongruence between vision and vestibular sensation could be reduced by these tactile sensations. Our results suggest that the effect of vibration on VR sickness depends on the participant and would be less for participants with severe sickness.

Observing optic flow that differs from the actual posture was a concern for exacerbating VE sickness due to the conflict between vision and vestibular sensation^56–59^, but in reality, there were no significant differences between conditions, indicating that it may not be as problematic as anticipated. However, although the Oculomotor scores were not significant, they were higher in the lying condition than in the standing or sitting conditions, suggesting the possibility of effects due to visual-vestibular conflict.

### Limitations and future research

We compared the synchronous vibrations with the asynchronous vibrations to test the effect of walking-simulating vibrations to the feet on the walking-related sensations and the embodiment. We did not include no-vibration condition. This is a limitation of the experiment. We had assumed that the actual walking posture is similar to the standing posture of the whole body compared to sitting posture, without considering the difference in the gravity of the head. However, in addition to the difference in gravity, whether the feet touch the ground or not is also important. The asynchronous foot vibrations as well as the synchronous vibrations may provide the pseudo-ground touching sensation in our experiment. Therefore, we should compare the synchronous and asynchronous vibration conditions with the no vibration condition in the future study.

In terms of the observation posture, the present study compared standing, sitting, and lying positions. The lying position involves a mismatch in the direction of gravity, while the sitting posture presents a postural mismatch, especially for legs. We have not investigated postures that are intermediate between these, or completely different postures such as inversion. It is a limitation. The direction of gravity is perceived by the vestibular system, and postural congruence is perceived by the proprioceptive system. Further research is necessary to quantitatively investigate how the congruence and conflict between each sensory system and vision affect the sensation of virtual walking.

The participants in this study had a gender ratio of 7:3 (male:female) and an average age of 22.38 years. The group was slightly male-biased and skewed towards the age group of young students. Therefore, it is not confirmed that our findings are valid for older age groups. Additionally, since the need for flexible observation postures due to physical constraints is expected to be higher in older age groups, it would be desirable to add age as a factor when considering the effects of observation postures in future studies.

Regarding physical characteristics, this study changed the avatar for men and women and aligned the height of the viewpoint from the avatar. There was a concern that if the avatar’s height greatly differed from the participant’s height, the body might feel like it was floating or submerged. The average height of the participants was 167.7 cm compared to the avatar height of 170 cm, so there were no remarks about discomfort due to height differences from the participants. However, in dealing with embodiment, the degree of congruence between the avatar and the participant’s physical characteristics may affect the evaluation of embodiment. Therefore, using avatars that are closer in size and appearance to the participants is ideal.

In the present study, participants did not move body parts such as legs or feet. Thus, there was a mismatch between the observed stimuli and the actual kinetic movement of the participants. It is interesting to investigate the effect of posture and foot vibrations on walking and embodiment sensations when participants move their feet or hands slightly. If the small movements, such as 10 mm, enhance the walking and embodiment sensations, it would be useful for improving our virtual walking system.

This research will contribute to comparing experiences and investigating appropriate stimulus presentation when using VR in more flexible postures, such as lying down, beyond the currently common standing or sitting positions. Walking-based spatial navigation is a universal task, and embodiment in virtual environments is an important factor in the increasingly popular metaverse. The sensations obtained from the physical body in the real environment often do not match the visual information obtained from observing the virtual environments. Thus, it is important to continue to consider what kind of additional stimuli and coordination with multisensory information are desirable to achieve a sense of immersion.

## Methods

### Participants

Twenty volunteers participated in the experiment (14 males and 6 females, mean 22.38 years old with 1.253 standard deviation). The sample size was determined by a power analysis: a medium effect size f = 0.25, alpha = 0.05, power = 0.8, and repeated measures of analysis of variance (ANOVA), three posture conditions $\times$ two vibration conditions, using G*Power 3.1^60,61^. All participants had normal binocular vision and physical abilities. They gave written informed consent before the experiment. The methods of the experiment were approved by the Ethical Committee for Human-Subject Research at the Toyohashi University of Technology, and all methods were performed in accordance with the relevant guidelines and regulations.

### Apparatus

The experimental environment was created and controlled by a computer equipped with Intel Core i7 10700, NVIDIA GeForce RTX 2070 Super, DDR4 32GB, and the Unity (2020.3.20f1) software. The visual stimuli were presented using a head-mounted display (HMD, HTC Vive Pro Eye, 1440 [width] $\times$ 1600 [height] pixels for each eye, refresh rate of 90 Hz).

**Figure 4:**
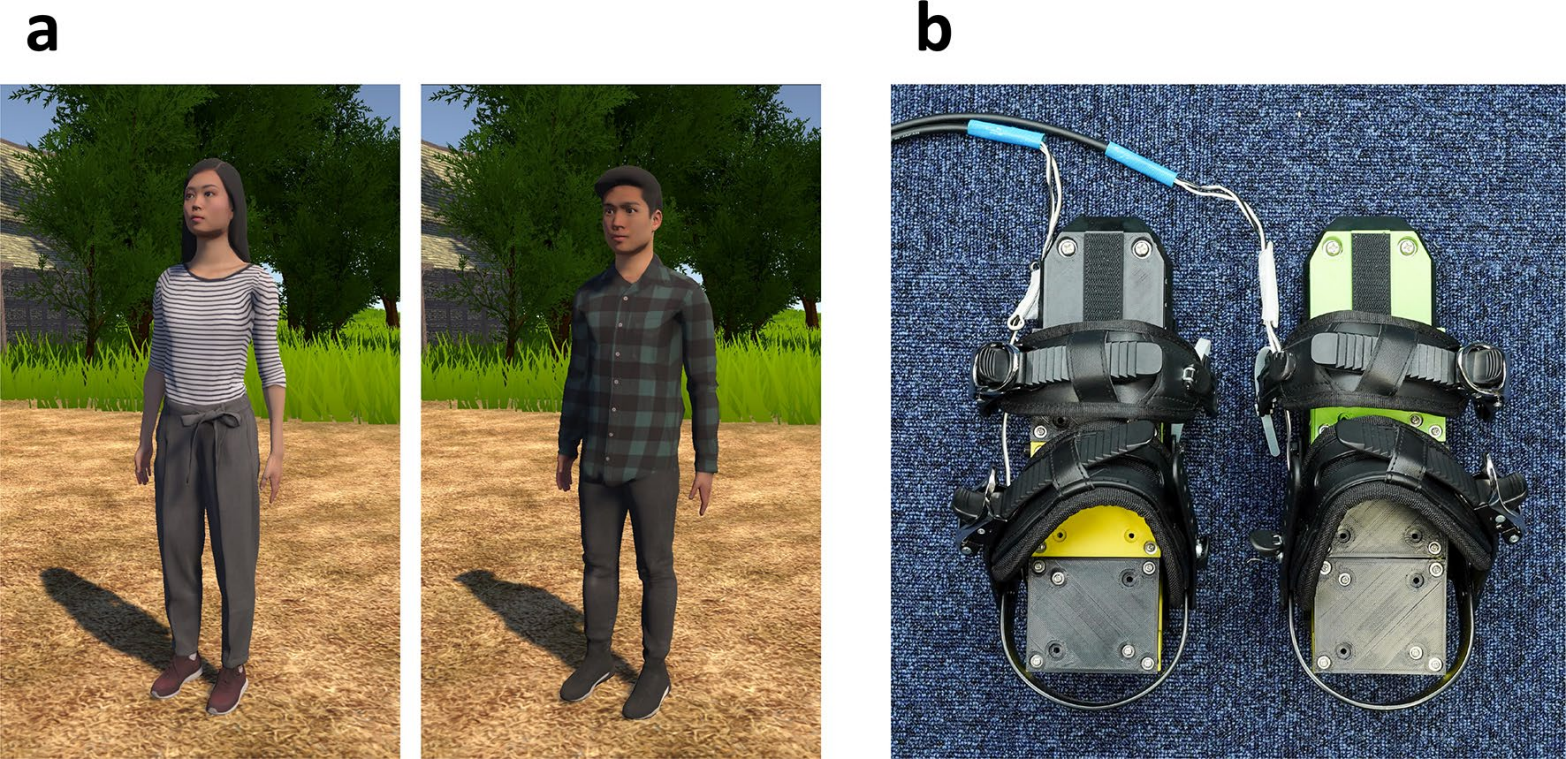
(**a**) Two types of avatar. (**b**) Foot vibration system device.

Participants’ avatars were either male (Toshiro Rigged 007, Renderpeople) or female (Rin Rigged 002, Renderpeople), based on their gender. The height of each avatar was set at 170 cm. Participants observed from a first-person perspective of the avatar, with the viewpoint height aligned for each avatar. Participants could look around while wearing an HMD, and rotation information was reflected in their view. However, position coordinates were not reflected to avoid changes in viewpoint height and conflicts with the avatar’s posture (Fig. 4a).

Tactile stimuli were presented at left and right forefeet and heels using four vibro-transducers (Acouve Lab Vp408). These transducers were affixed to a 3D-printed ABS plate and supported by four springs, with four units used for each forefoot and heel. An additional unit was used to support the middle foot of the participant, without carrying a transducer. The transducer units were mounted on a modified exterior of ski boots, and the participant’s feet were secured to the device using two fixtures (Fig. 4b).

The transducers were powered by a multichannel power amplifier (Behringer EPQ304, 40W $\times$ 4/8$\Omega$) and an audio interface (Focusrite Scarlett 18i20), with ASIO utilized for the driver interface. The amplitude of vibration was maintained at a fixed level throughout the experiment, and was found to be sufficiently strong to elicit the desired sensations even when participants wore socks. To minimize auditory distractions, participants wore headphones during the experiment, which featured noise cancellation functionality to reduce the impact of external sounds. In addition, white noise (70 dBA) was presented during stimulus presentation to mask any remaining auditory stimuli.

### Stimuli and conditions

#### Stimuli

**Figure 5:**
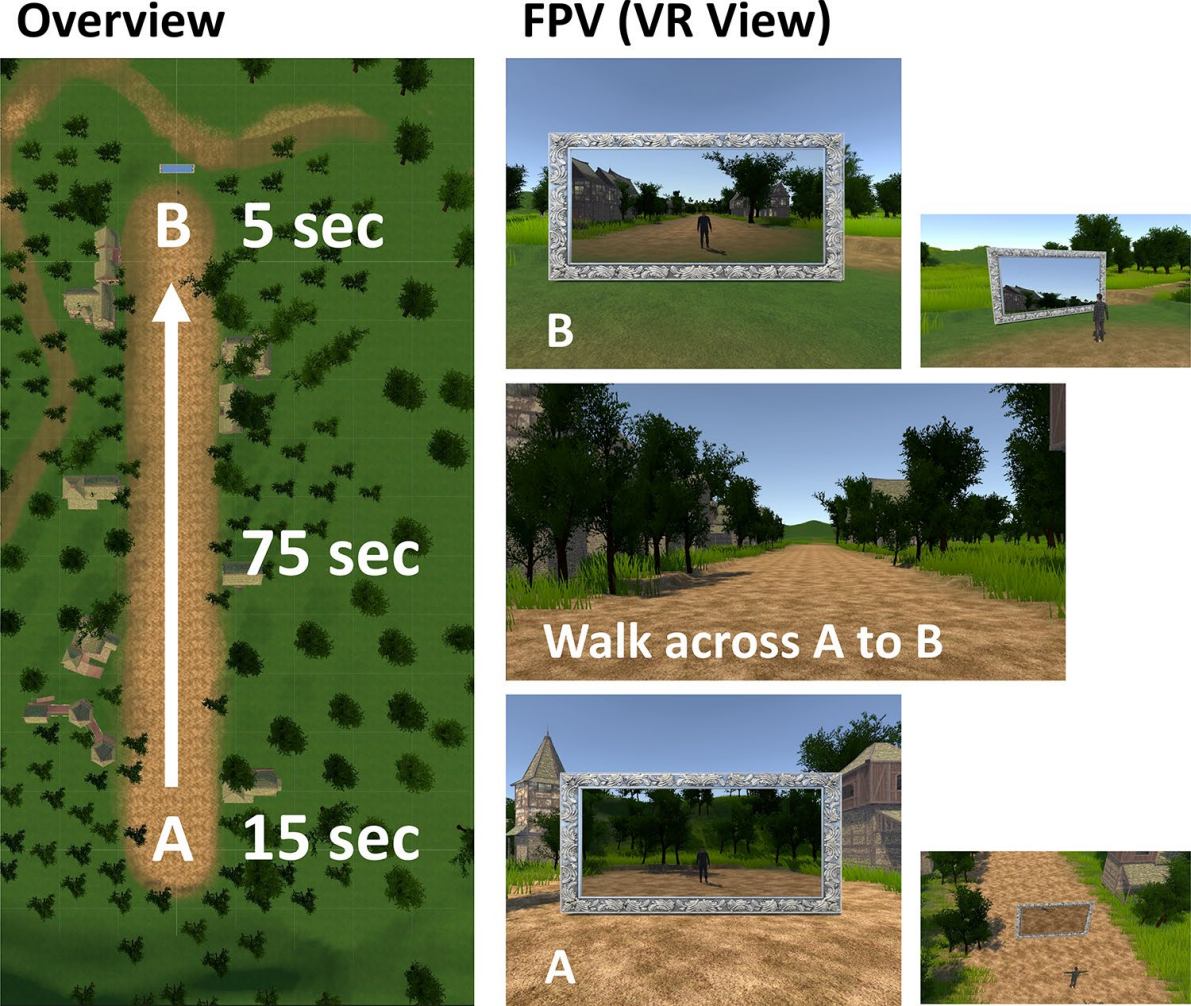
Experimental virtual environment. The avatar walked from point A to point B (Overview). Example scenes from the participant’s point of view (*FPV* first-person view).

A terrain and structure-filled area (250 m [width] $\times$ 250 m [depth]) was created, and a flat walk-able area (10 m [width] $\times$ 120 m [depth]) was set in the center. Participants walked across this flat area from one end to the other. Two mirrors (2.5 m [width] $\times$ 1.25 m [height], 4 m in front of the avatar’s position) were placed at the starting and ending points. The mirror at the starting point was displayed for 15 s from the time the screen switched-on and removed when the walking began. The avatar walked forward for 75 s (5.16 km/h, 2.20 steps/s). The mirror at the end point was displayed for 5 s as soon as the walking ended. Participants observed the scene from the avatar’s viewpoint (first-person view) (Fig. 5).

The foot stimuli were made by recording the sound of walking in sneakers. A single step was cut out from the recorded data, to make the sound clip of 500 ms. This clip was used for the heel and forefoot with 100 ms gap.

#### Conditions

**Figure 6:**
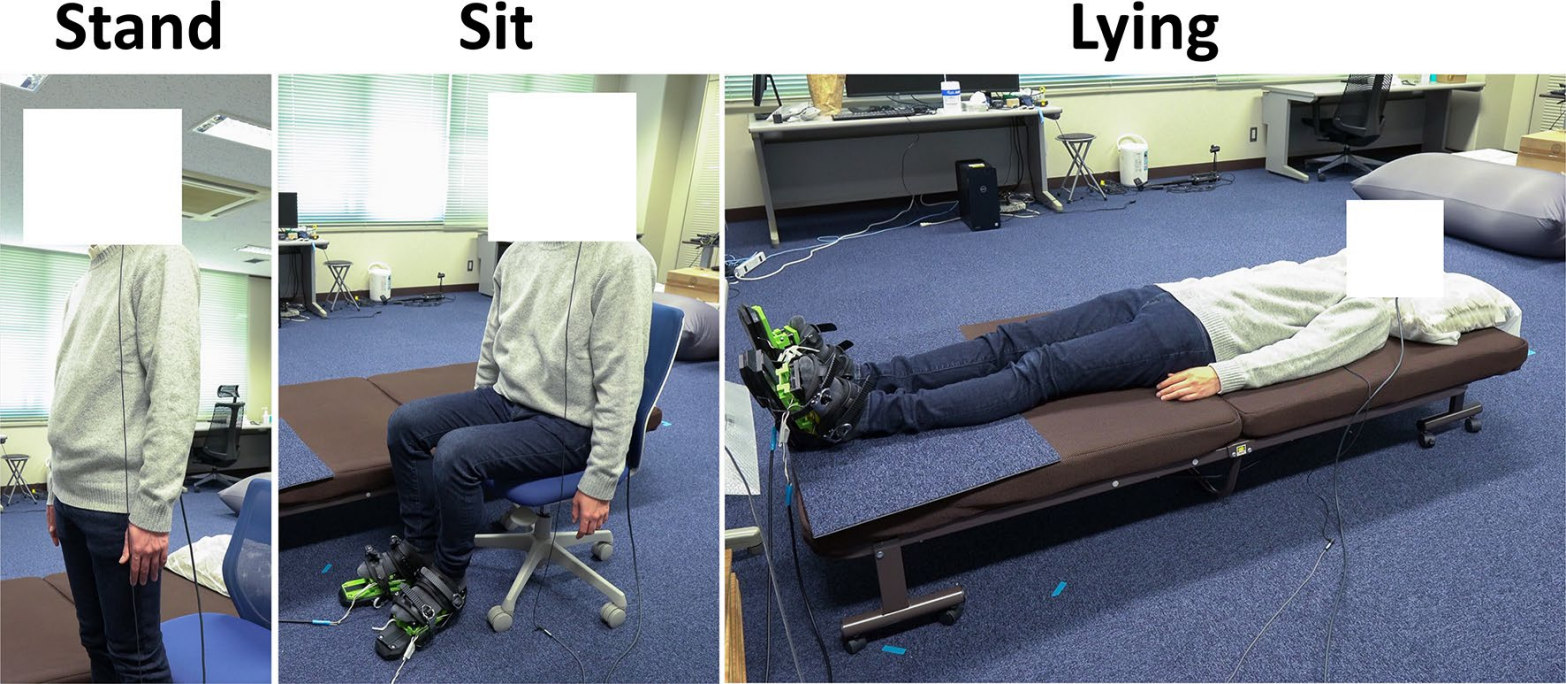
Posture conditions. Participant performed the experiment during standing, sitting or lying.

Combinations of 3 levels of posture condition (standing, sitting, lying) (Fig. 6) and 2 levels of foot vibration condition (synchronous, asynchronous) with within-subject design were used. The participants observed 18 trials (3 posture conditions $\times$ 2 vibration conditions $\times$ 3 repetitions) in random order. For the standing and sitting conditions, the axis of the participant’s trunk and the vertical axis in the virtual environment were consistent, while for the lying on one’s back condition the vertical axis in the virtual environment was rotated 80 deg to align the directions of the participant’s and the avatar’s trunks approximately. For foot vibration, the synchronous condition presented vibrations synchronously with the avatar’s walking animation, and the asynchronous condition presented the same number of vibrations randomly. We employed the asynchronous vibrations instead of the no-vibration for the control condition because the previous study^62^ compared the synchronous, asynchronous, and no vibration conditions, and showed that the synchrony of the vibration is critical for the virtual walking.

### Questionnaire

All questionnaires were presented on the screen of the HMD, and participants used a computer-mouse to respond to the questionnaire items. The mouse was provided to participants when the scene transitioned from the stimulus presentation scene to the questionnaire scene, and was collected upon completion of the questionnaire responses.

#### Walking-related sensation and telepresence

Participants were asked to rate their experience of walking-related sensations, such as the feeling of movement in their legs. They were also asked to rate their experience of telepresence, which refers to the sense of being present in a virtual environment.

To ensure that the ratings were consistent across participants and trials, the rating items were based on previous studies^26,39,62^ that have investigated the subjective experience of walking and telepresence. These items were carefully selected to capture the most relevant aspects of each sensation to test the hypotheses H1a and H1b, and were designed to be easy for participants to understand and use. We measured telepresence even though it was not mentioned in the hypotheses because the enhancement of embodiment promotes telepresence in physical and virtual environments and it is one useful measure of participants’ virtual walking experience.

To minimize any potential biases that could arise from the order in which the rating items were presented, the order of these items was randomized in each trial. This ensured that each participant rated each item in a different order, reducing the likelihood of any order effects influencing their responses.

1. I felt as if my whole body was moving forward (self-motion).
2. I felt as if I was walking forward (walking sensation).
3. I felt as if my feet were striking the ground (leg action).
4. I felt as if I were actually there in the scene (telepresence).

**Figure 7:**
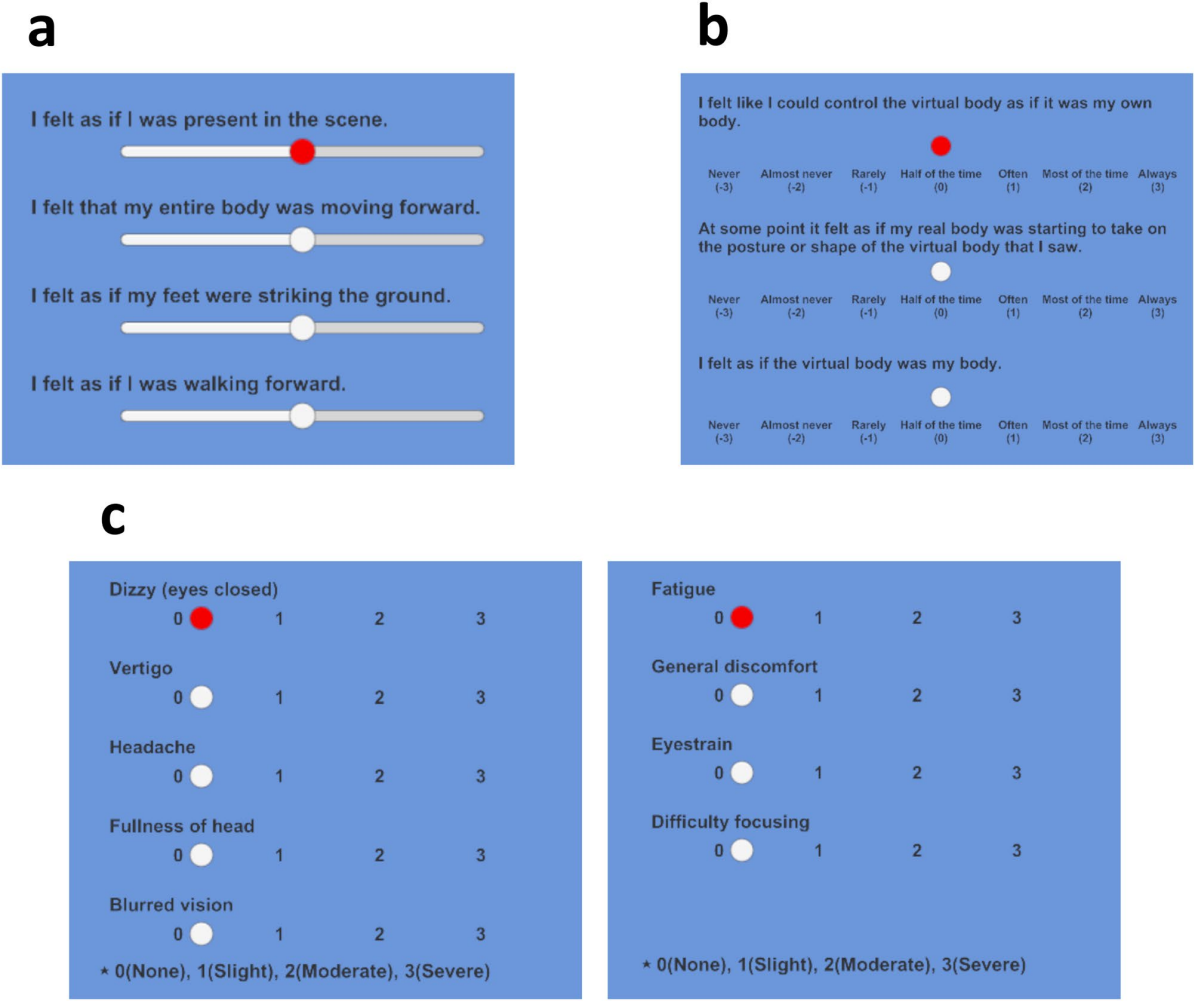
The screens of the questionnaires. (**a**) Walking-related questionnaire. (**b**) Embodiment questionnaire. (**c**) VRSQ questionnaire. The red dot means the choice of the currently active item.

Participant responses were gathered utilizing a visual analog scale (VAS). The VAS consisted of a horizontal line displayed on a screen, with the leftmost endpoint representing the absence of the sensation in the item sensation and the rightmost endpoint representing an experience equivalent to actual walking. The responses were then digitized for analysis, using a scale of 0 to 100 (Fig. 7a).

#### Embodiment score

To assess the subjective evaluation of embodiment for the Avatar, the Peck et al.^63^.questionnaire was employed. The questionnaire consisted of three items, namely posture matching, body ownership, and sense of agency, which were selected to align with the evaluation of walking-related sensations in the previous section. Posture matching was adopted from item R5 of the original questionnaire, body ownership from item R10, and sense of agency from item R13. Participants provided their ratings using a 7-point Likert scale, with the presentation order of the items being randomized to minimize any order effects (Fig. 7b).

1. At some point it felt as if my real body was starting to take on the posture or shape of the virtual body that I saw. (posture)
2. I felt as if the virtual body was my body. (body ownership)
3. I felt like I could control the virtual body as if it was my own body. (sense of agency)

#### Virtual Reality Sickness Questionnaire (VRSQ)

The Virtual Reality Sickness Questionnaire (VRSQ)^64^ was utilized to evaluate the occurrence of sickness in VR. The VRSQ comprises 9 questions that are used to calculate the Oculomotor and Disorientation indicators. Participants rated their responses on a 4-point Likert scale, and the order of presentation of the questions was randomized to minimize any potential order effects (Fig. 7c). Although the VRSQ questionnaire is not directly related to the research question, the discrepancy between visual and somatosensory sensations in the VR experience may cause sickness, and it is important to investigate the actual effects needs to be evaluated including the effect of foot vibrations.

### Procedure

Participants wore the HMD, the foot vibration devices, and the headphone. An instruction for the posture was displayed on the HMD when the experiment began. After participant’s changed the posture, a fixation point was displayed (5 s), then the trial scene started. During the trial, the participants looked at the avatar in the mirror (15 s) to increase the illusory ownership of avatar body, observed walking (75 s), and stood still in front of the mirror again (5 s). Then, the walking-related questionnaire was displayed until they responded. The order of items was randomized in each trial. The embodiment questionnaire and the VRSQ followed it.

### Image consent and authorship

The photograph depicted in Fig. 6 was taken by the author, Junya Nakamura, who is also the subject of the image. The author consents to the use of this image in the manuscript.

## Data Availability

All data generated or analyzed during this study are available on request to the corresponding author.
